# On-chip non-volatile all-optical residual neural network accelerator

**DOI:** 10.1038/s41377-026-02325-2

**Published:** 2026-08-19

**Authors:** Zhiqiang Quan, Bing Han, Xiaoxiao Ma, Jingyuan Qu, Xiangshui Miao, Qiang He, Jian Wang

**Affiliations:** 1 Wuhan National Laboratory for Optoelectronics and School of Optical and Electronic Information, Huazhong University of Science and Technology, Wuhan, 430074 Hubei China; 2 Hubei Optical Fundamental Research Center, Wuhan, 430074 Hubei China; 3 Optics Valley Laboratory, Wuhan, 430074 Hubei China; 4 School of Integrated Circuits, Huazhong University of Science and Technology, Wuhan, 430074 Hubei China

**Keywords:** Integrated optics, Silicon photonics

## Abstract

Optical neural networks (ONNs) hold substantial potential in artificial intelligence, promising faster processing speed and reduced energy consumption compared to traditional electronic neural networks, by implementing matrix operations with optical computations. Current ONN architectures predominantly rely on single- or multi-channel convolutions to accelerate computing operations. However, high-performance neural networks, such as ResNet-50, SSD, and Transformer, employ residual convolutions for deep feature extraction instead of single- or multi-channel convolutions. To make ONNs widely functioned in deep neural networks, we propose the Non-Volatile All-Optical Residual Convolution Accelerator (NARCA), based on phase change material (PCM), which weight update energy consumption is only 9.8 μW, much smaller than that of thermal-optical unit with about 10 mW. The NARCA remain high convolution precision, and the experimental results show that the NARCA-based architecture outperforms the conventional optical convolution architecture across different neural-network tasks. Moreover, we demonstrate 128 GHz high-speed optical residual convolution, which greatly improves the residual convolution operation speed compared with the electrical architecture, with a relative root mean square error (RMSE) of less than 0.125.

## Introduction

Optical computing shows significant advantages over traditional electrical computing, offering unparalleled speed, vast bandwidth, and exceptional energy efficiency. Unlike electronic circuits constrained by data transmission and processing speed, optical systems transmit and process information at the speed of light, while leveraging the orthogonality of wavelength, polarization, and mode to perform parallel computations. This potential allows for rapid and efficient computation of large-scale tasks^1–3^. These unique advantages have garnered widespread attention for optical computing, particularly within the realm of artificial intelligence (AI).

Recent researches have demonstrated that specialized optical system architectures can effectively perform specific operations in traditional electronic neural networks^4–7^. For instance, matrix-vector multiplication is implemented using optical Mach-Zehnder interferometer (MZI) architectures^8–10^, multi-channel convolution operations are executed via micro-ring resonator arrays^11–13^, and nonlinear mappings are realized through integrated optical waveguides with two-dimensional materials^14–16^. These developments prove the potential of optical neural network architectures to surpass electronic computing in terms of speed and energy efficiency while maintaining computational accuracy. However, most optical neural network architectures lack the ability to dynamically reconfigure weights. Typically, the weights of an optical neural network are pre-calculated and fixed on specific optical computing units, such as diffraction phase elements, and remain unchanged throughout the computation^17–20^. This rigid approach limits the adaptability of optical neural networks to specific tasks. Furthermore, some studies have attempted to achieve adjustable weights for optical convolution and fully connected operations using thermo-optic micro-rings or phase shifters. However, these modulation methods suffer from low weight configuration speeds (on the order of kHz)^8–10,12,13,21–23^. As a result, there is an urgent need for a reconfigurable optical neural network architecture that can rapidly adapt to various computational demands, thus enabling more efficient applications and broader operability.

A promising solution to this challenge is the use of phase-change materials (PCMs) to construct high-speed, energy-efficient, and highly integrated optical neural networks^24–26^. This is due to the ability of PCMs to undergo reversible and rapid phase transitions (in nanoseconds) between crystalline and amorphous states by electrical or optical pulses, enabling ultrafast modulation of light phase or intensity^27–35^. Additionally, the non-volatile nature of PCMs (where the crystalline or amorphous state is maintained without external power) results in optical devices based on PCMs that exhibit extremely low computational energy consumption. To date, PCM-based optical synapses have demonstrated high-speed, low-energy optical modulation, providing a foundation for energy-efficient, reconfigurable optical neural networks capable of performing complex tasks. Optical pulse neural networks and optical convolutional neural networks based on PCMs have successfully implemented convolution and nonlinear activation operations typically based on electronic neural networks, achieving high computational performance. However, with the evolution of AI algorithms in large models, such as ResNet-18, ResNet-50, and Transformer networks^36,37^, residual convolution has demonstrated significant advantages over traditional convolution operations in neural networks, particularly in improving training efficiency and enabling deeper network architectures^38^. To address this limitation, we propose the Non-Volatile All-Optical Residual Convolution Accelerator (NARCA), a novel optical computing architecture based on PCM. NARCA is capable of constructing deep residual neural networks, thus enabling more efficient and flexible optical neural network computation.

In this paper, we design and fabricate the on-chip NARCA using phase change material (Ge_2_Sb_2_Te_5_, GST), capable of performing residual convolution operations in the optical domain. The residual neural network based on NARCA shows greater performance compared to the traditional optical network in different neural network tasks, 17% classifacation accuracy improvement in image classification task and 0.18 structural similarity index (SSIM) value improvement in image denoising task. Moreover, we demonstrate 128 GHz ultrafast optical residual convolution, a significantly higher hardware convolution operation speed compares with the electrical architecture. Experiments based on NARCA show that the errors between optical and electrical residual convolution calculations are small. The results indicate that the RMSE of the image residual convolution results between the two methods (optical residual convolution based NARCA, electrical residual convolution) is less than 0.125, allowing for a near-complete recovery of image information. Therefore, NARCA can effectively improve the deep optical neural network performance compare with the traditional optical convolution architecture, and replace the residual convolution module in electronic neural networks, thereby promoting the application of optical computing in more complex and generalized neural network models.

## Results

### Design and fabrication of the NARCA

**Fig. 1:**
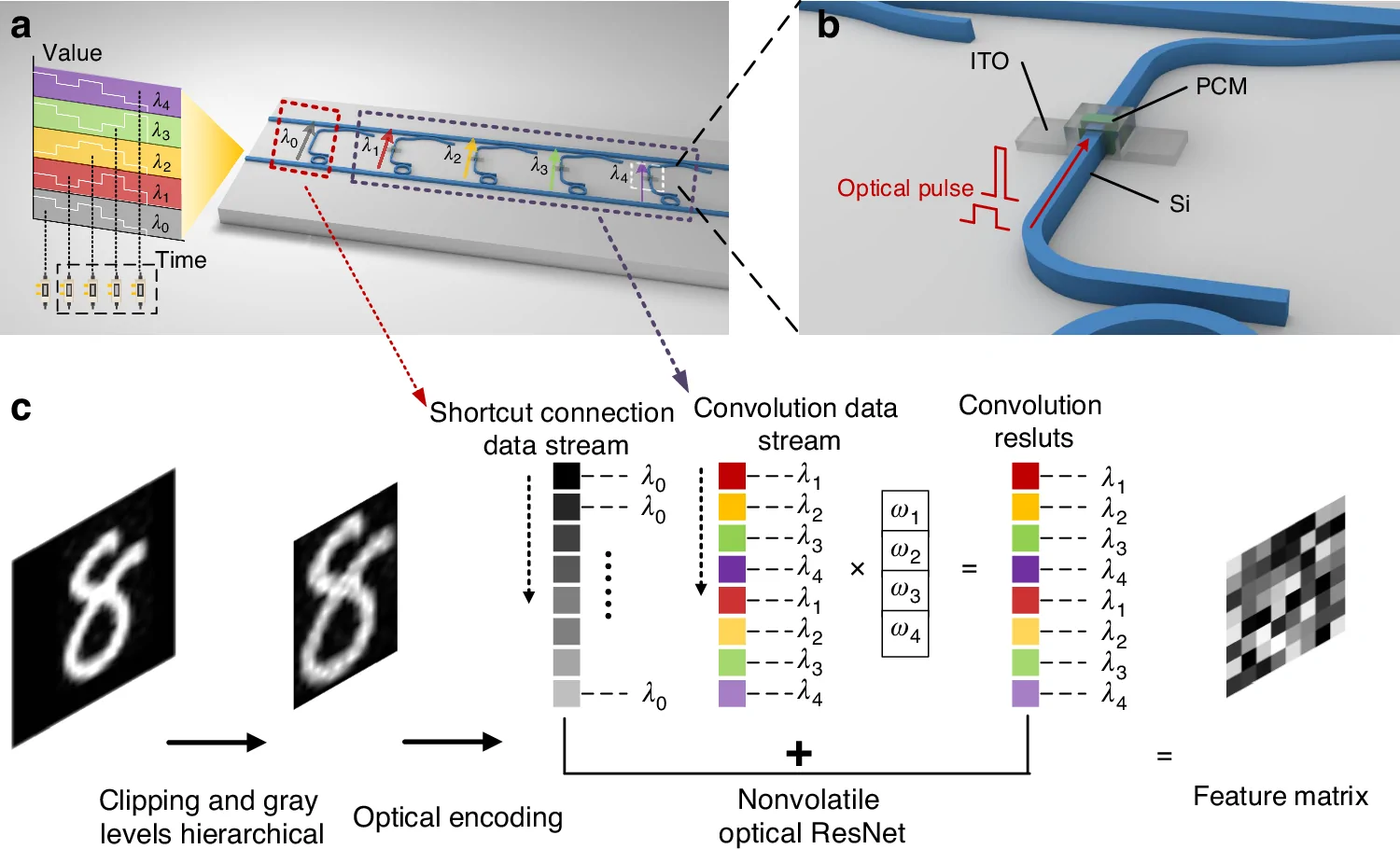
Schematic diagram illustrating the principle of optical residual convolution. **a** The NARCA architecture on the SOI platform. The input matrix is optically encoded using five wavelengths (*λ*₀, *λ*₁, *λ*₂, *λ*₃, *λ*₄), where λ₀ encodes all pixels as the shortcut connection data flow, and *λ*₁, *λ*₂, *λ*₃, *λ*₄ encode the convolutional regions as the convolution data stream. An on-chip filter array separates the shortcut connection data stream from the convolution data stream, while an on-chip coupler combines the post-convolution data stream with the shortcut connection data stream, forming the residual block. The size of the convolution kernel is 2×2. **b** On-chip photonic synapse. PCM and indium tin oxide (ITO) capping are deposited on the waveguide sequentially, with the weights adjusted by loading different optical pulses. **c** Schematic diagram of the data processing flow for image feature extraction using optical residual convolution. After the input image matrix undergoes clipping and grayscale processing, WDM encoding is applied. The shortcut connection data stream encodes each pixel of the image, while the convolution data stream encodes the convolutional pixels of the image matrix using four wavelengths. The multi-wavelength convolution results are added to the single-wavelength shortcut connection data stream to obtain the feature matrix information of the image

Similar to traditional electronic residual neural network architectures, optical residual neural networks require the incorporation of shortcut connections during convolution to mitigate feature loss in the computing process. In this study, an optical residual convolution accelerator is proposed based on the principles of optical incoherent computation, as illustrated in Fig. 1a. The input data is encoded via wavelength-division multiplexing (WDM) using five distinct wavelengths. The wavelength *λ*₀ encodes each pixel of the input image, forming the shortcut connection data stream, while the other four wavelengths (*λ*₁, *λ*₂, *λ*₃, *λ*₄) encode the convolutional information of the input data, forming the convolution data stream. These five wavelength signals are coupled to the on-chip NARCA through a wavelength-division multiplexer.

Since the shortcut connection data stream does not require linear multiplication, while the convolutional data stream necessitates configurable linear multiplication, the on-chip NARCA must effectively separate these two data streams. In this study, we employ five micro-ring resonators operating at different wavelengths to separate the shortcut and convolution data streams. Each micro-ring resonator couples one of the five wavelength signals into distinct transmission channels. Four of these channels are equipped with non-volatile all-optical synapses, whose weight configuration is adjustable. The non-volatile all-optical synapse operates by adjusting the crystallization ratio of PCM through on-chip optical excitation, which leads to changes in its optical response. When specific wavelength signals (*λ*₁ to *λ*₄) propagate through the waveguide region with PCM, the greater optical loss in the PCM, the higher transmission optical loss of the waveguide. This results in modulation of the optical intensity response, thereby enabling multiplication operations. Meanwhile, the capacity to maintain the crystalline configuration without the necessity for external energy after the initial modulation renders these optical synapses highly energy efficient.

The structure of the non-volatile all-optical synapse is illustrated in Fig. 1b. A thin PCM film is deposited and patterned on the silicon waveguide. By altering the crystallization state of the PCM, the propagation loss in the waveguide region can be controlled. This is achieved by encoding the optical pulses with varying pulse energy and pulse width, which in turn alters the optical absorption by the PCM. The change in absorbed energy results in a modification of the crystallization state of the PCM, thereby enabling the encoding of waveguide transmission loss. This mechanism facilitates the adjustment of the non-volatile all-optical synapse weights, resulting in the control of the optical computing results. The optical encoding method for the input data is illustrated in Fig. 1c. The original image is preprocessed through clipping and grayscale transformation to generate the input image data for further processing. In NARCA, four all-optical synapses form a 2 × 2 convolution kernel, which performs convolution on four wavelength signals (*λ*₁, *λ*₂, *λ*₃, *λ*₄). The convolution result is then combined with the optical signal at wavelength *λ*₀, which represents the shortcut connection data flow, using a broadband directional coupler (BDC). The image is first preprocessed through clipping and grayscale transformation to obtain the input image data. This data is then encoded using five independent electro-optic modulators (EOMs), while each corresponding to one of the five wavelengths *λ*₀_-4_. The signal at *λ*₀ performs traversal encoding of the entire input image, while the signals at *λ*₁, *λ*₂, *λ*₃, and *λ*₄ encode specific regions of the image for convolution via WDM. The signals at *λ*₁, *λ*₂, *λ*₃, and *λ*₄ are multiplied with the corresponding on-chip convolutional kernels (*ω*₁, *ω*₂, *ω*₃, *ω*₄). These results are then added to the λ₀ shortcut connection signal, producing the feature matrix for the image. The calculation process is given in the following equation:

$$y=x\left({\lambda }_{0}\right)+\left[\begin{array}{cc}x\left({\lambda }_{1}\right) & x\left({\lambda }_{2}\right)\\ x\left({\lambda }_{3}\right) & x\left({\lambda }_{4}\right)\end{array}\right]* \left[\begin{array}{cc}{\omega }_{1} & {\omega }_{2}\\ {\omega }_{3} & {\omega }_{4}\end{array}\right]$$

Hear, *x* represents the initial signal intensity, * is the convolution symbol.

**Fig. 2:**
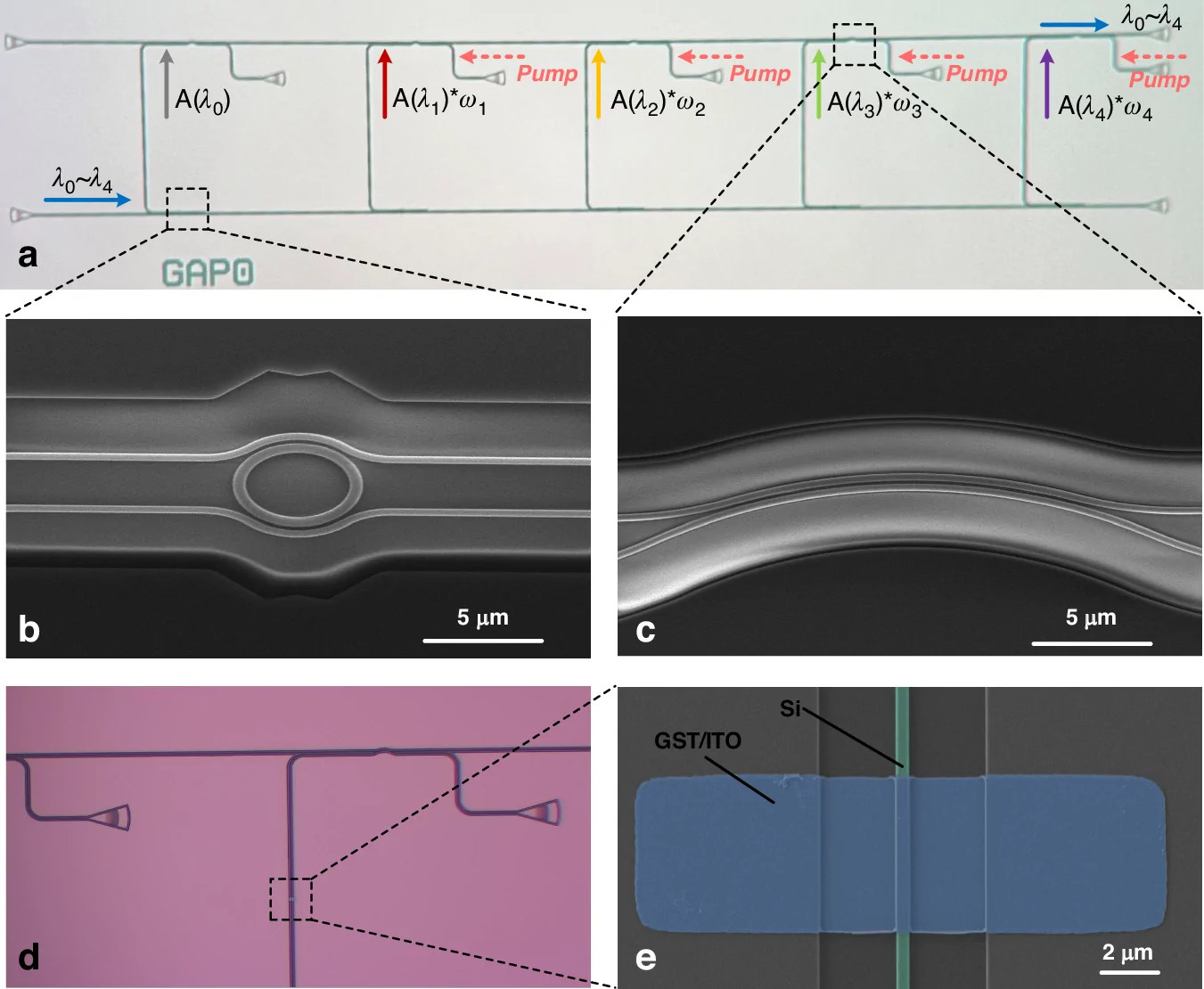
Schematic of the NARCA device and structural characterization images. **a** Optical microscope image of the NARCA architecture. Five wavelength signals pass through micro-ring resonators with different resonance wavelengths, transmitting through various channels. The output signal intensity from four of these channels can be adjusted via non-volatile all-optical synapses. Different wavelength signals are coupled into the output channel via the BDC. **b**, **c** SEM images of the micro-ring resonators and BDC. Optical microscope image of the non-volatile all-optical synapses (**d**) and its magnified details (**e**). The non-volatile all-optical synapses, where GST and ITO are deposited on the waveguide. The crystallization state of the GST is controlled by adjusting the optical pulse energy in the waveguide, and the wavelength of the pump laser is 1549.988 nm

Figure 2 shows the measured optical and scanned electron microscope (SEM) images of the fabricated NARCA based on the silicon-on-insulator (SOI) platform. In Fig. 2a, the wavelength-division multiplexed signal of the encoded input image data couples into NARCA through a vertical grating coupler. Different wavelengths (*λ*₀ to *λ*₄) pass through micro-ring resonators with different resonance wavelengths and are transmitted through specific channels (Channel 1 to 5), with their initial intensities represented as A(*λ*ᵢ) (where *i* ranges from 0 to 4). When the non-volatile all-optical synapses adjust the transmission losses of specific waveguide channels, the output optical intensity is also modulated. For optical signals at wavelengths *λ*_i_, the impact of the non-volatile all-optical synapse on the signal intensity is denoted as ωᵢ. Therefore, the output intensity can be expressed as A(*λ*ᵢ)*ωᵢ. In the NARCA, four wavelengths are used for WDM encoding of the convolutional regions. Thus, the array of non-volatile all-optical synapses consists of 1×4 synapse units, which is treated as a 2×2 kernel convolutional filter. The convolved signal is combined with the shortcut connection signal A(*λ*₀) through a BDC into the same output channel. Since each channel operates at a different wavelength, the output optical intensity is the sum of the convolved signal intensity and the shortcut connection signal intensity, thus realizing the optical residual convolution functionality. Figure 2b and c shows the SEM characterization images of the fabricated micro-ring resonators and the BDC, with detailed broadband transmission results provided in Section 1 (Supplementary Materials). Figure 2d displays a microscopic image of the non-volatile all-optical synapses in NARCA. Figure 2e presents a magnified view of the non-volatile all-optical synapse, where the PCM GST is deposited on the silicon waveguide. An ITO layer is applied on top of the GST to prevent oxidation during the phase transition of PCM layer. When the optical signal passes through this waveguide region, the GST, having a higher real and imaginary refractive index compared to silicon, causes the optical signal to be absorbed. As a result, when the optical pulse energy is relatively large, the temperature of the region with GST will rise significantly due to the absorption of optical energy. By adjusting the pulse width and amplitude, the crystallization ratio of GST can be finely controlled, which in turn influences the transmission loss of the waveguide. Here, the length of the GST along the propagation direction of the optical signal is 5.6 μm, with a thickness of 25 nm, while the ITO layer has the same planar geometry as the GST and a thickness of 20 nm. The impact of the GST geometry on the transmission characteristics of the optical waveguide is detailed in Fig. S3 of the Supplementary Materials.

**Fig. 3:**
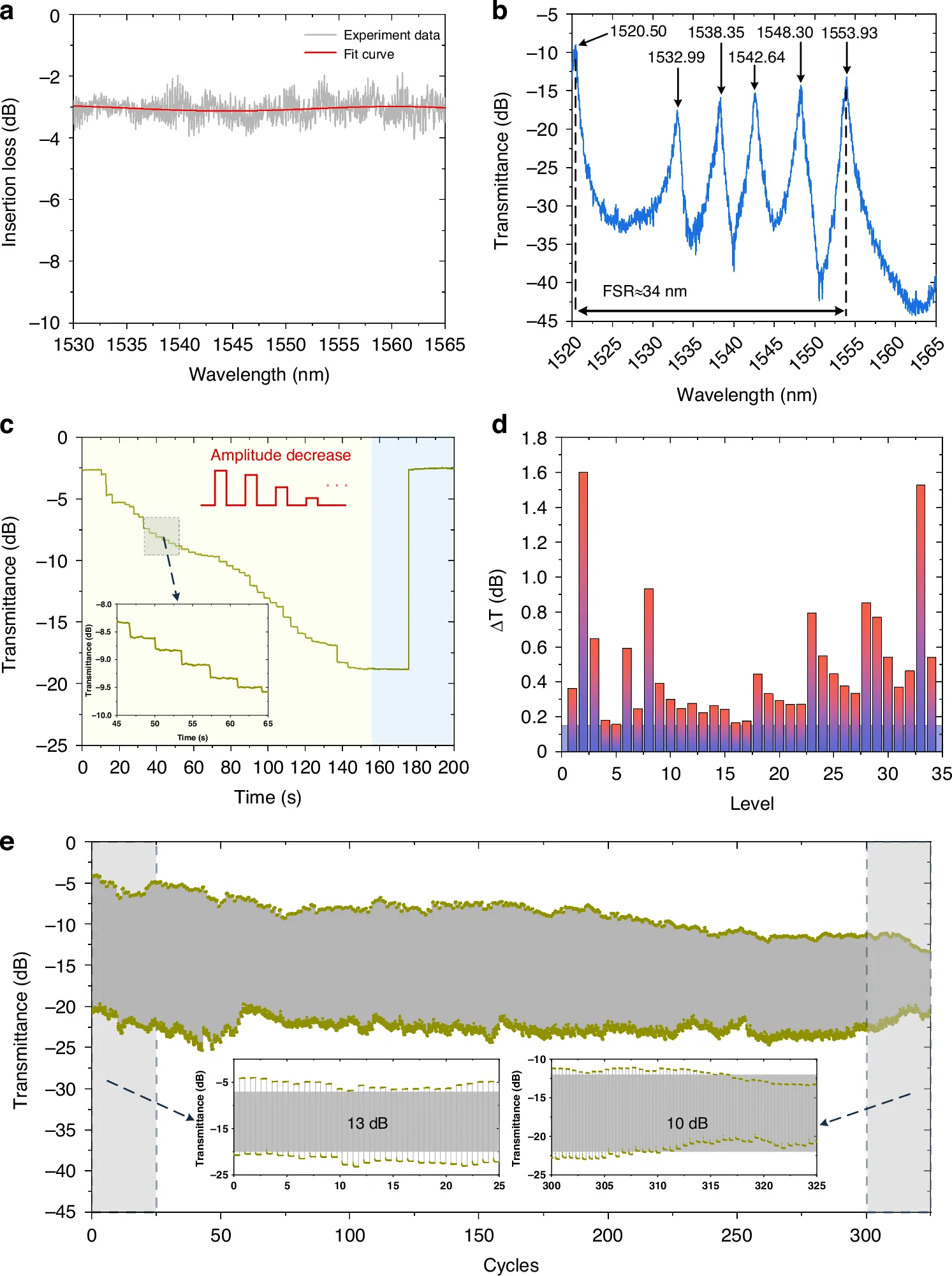
NARCA optical performance measurement. **a** Insertion loss of the non-volatile optical synapse, where the gray curve represents the measured data, and the red curve represents the fitted result. **b** Normalized transmission spectrum of NARCA after removing grating losses. **c** Multi-bit weight encoding of the non-volatile all-optical synapse, achieving more than 34 levels (>5 bits) of encoding. **d** Optical intensity difference between adjacent weight levels, with a minimum difference greater than 0.15 dB. **e** Cycling lifetime test of the non-volatile all-optical synapse, where the inset shows the extinction ratio of the switching states for the first 25 cycles and the last 40 cycles. The synapse achieves 300 cycles of switching with an extinction ratio greater than 10 dB

Figure 3a shows the insertion loss of the device with the deposition of GST film on the waveguide. Compared to the device without GST film, the transmission loss increases approximately 3 dB. This reduction is attributed to the light absorption of the GST in its amorphous state. Figure 3b illustrates the broadband output spectrum of the NARCA device. The spectrum is tested to select appropriate wavelengths for the carrier signals of both the shortcut connection and the convolution data streams. Each of the five micro-ring resonators, with distinct resonance wavelengths, exhibits good extinction ratio and isolation, with a free spectrum range (FSR) of approximately 34 nm. We chose five wavelengths in the C-band, 1532.99 nm, 1538.35 nm, 1542.64 nm, 1548.30 nm, and 1553.93 nm, for signal transmission in each channel. Among them, the 1553.93 nm wavelength carries the shortcut connection information in the optical residual convolution process, while the 1532.99 nm, 1538.35 nm, 1542.64 nm, and 1548.30 nm wavelengths carry the convolution information. It is noteworthy that the insertion loss varies with wavelength. To ensure consistent image signal strength across all channels, compensation for the insertion loss of different wavelengths is required. Using the insertion loss of the 1553.93 nm wavelength carrying the shortcut connection signal as the normalization standard, the four convolution signals need compensation of 4.48 dB, 2.74 dB, 2.27 dB, and 1.35 dB, respectively.

Figure 3c shows the results of a multi-level weight modulation test of the non-volatile all-optical synapse in NARCA (subtracting the broadband response of the device before deposition of the GST/ITO film). During the multi-level weight modulation process of the optical synapse, the pulse width of the pulsed laser remains constant at 150 ns, while the output peak power gradually decreases from 14.8 dBm to 8 dBm. By varying the energy of the pump light pulses, we demonstrate weight encoding with 34 levels (>5 bits). The inset illustrates the detailed process of weight modulation. To ensure that the optical intensity variations resulting from each weight modulation can be effectively detected by the photodetector, we measure the optical intensity differences between adjacent weight levels, as shown in Fig. 3d. It can be observed that the optical output intensity difference between adjacent weight levels exceeds 0.15 dB, which ensures that each weight level can be effectively distinguished by most optoelectronic detectors. Meanwhile, to investigate the endurance of the non-volatile all-optical synapse, we demonstrate a device “on/off” cycling test, as shown in Fig. 3e. The instantaneous optical powers required to crystallized and amorphized the GST are 17.1 dBm and 29.9 dBm, respectively, with corresponding pulse widths of 150 ns and 10 ns. It can be seen that the non-volatile all-optical synapse has achieved 300 cycles of switching with an extinction ratio greater than 10 dB. The inset shows magnified images of the first 25 and last 40 cycles. Initially, the transmission contrast exceeds 13 dB, and although the contrast gradually decreases over time. However, even after 300 cycles, the two transmission levels (amorphous state and crystalline state of GST) remain distinguishable. This indicates that the non-volatile optical synapse can undergo at least 300 reversible cycles with a switching ratio greater than 10 dB, ensuring the long-term stability of weight updates in NARCA.

However, In PCM-based optical synapses, the transition between crystalline and amorphous states is inherently stochastic due to the thermally driven nature of crystal nucleation and local material oxidation. This randomness introduces write noise, leading to slight deviations in the refractive index of even phase change materials programmed with the same pulse parameters, thus affecting the accuracy of synaptic weight encoding. As shown in Fig. 3e, this write noise can be clearly observed during the SET/RESET process due to the involvement of rapid quenching and high thermal gradients. This variation accumulates over multiple programming cycles, which in turn leads to noticeable weighting errors (the switching states are indistinguishable). To mitigate this problem, the pulse energy and duration of the SET and RESET operations have been carefully optimized to effectively reduce the resulting phase changes. As a result, the ‘0’ and ‘1’ states of the device remain well separated even after 300 modulation cycles.

It is worth noting that similar modulation errors are also present in other thermally tuned photonic neural network platforms such as MZI- and MRR-based architectures. To further suppress randomness during weight programming, we adopt a 5-bit discrete weight encoding scheme. Compared to high-resolution analog encoding, this approach enhances tolerance to small fluctuations and improves overall robustness. Fortunately, since synaptic weights are configured after offline training and do not require continuous refreshing or reprogramming during inference, the write frequency is inherently low. This further limit the impact of cumulative drift. Therefore, although write noise is intrinsic to PCM-based systems, our results indicate that it does not significantly degrade the long-term computational reliability of NARCA in neural network applications.

### Compare with traditional optical convolution

**Fig. 4:**
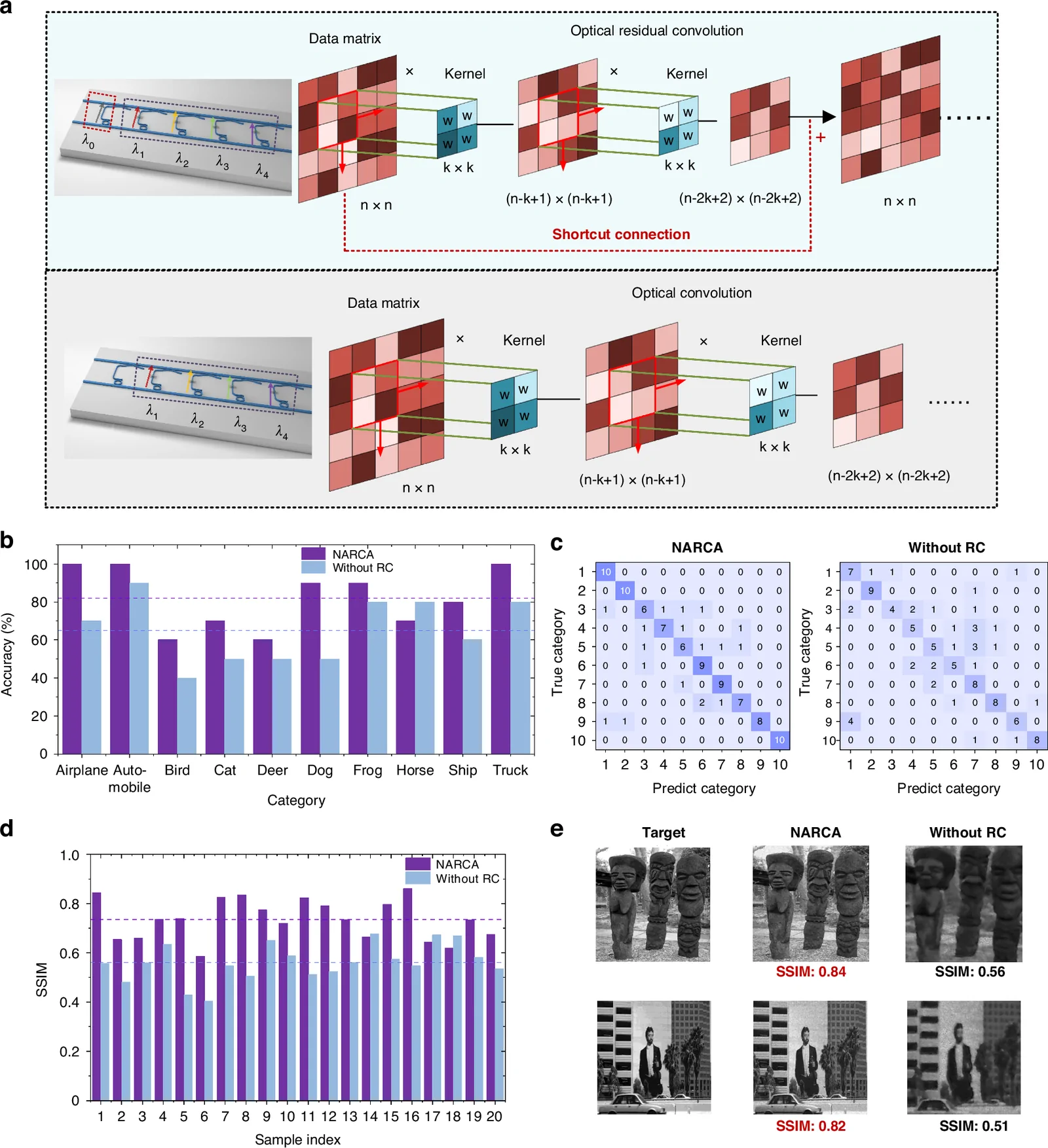
Performance comparison between optical neural networks with and without residual convolution (RC). **a** Schematic diagram of the NARCA-based optical neural network architecture and the conventional optical neural network without residual convolution (RC). **b** Classification performance of the two optical neural networks (with NARCA and without RC) on the CIFAR-10 dataset. The dashed lines with different colors indicate the average classification accuracies. **c** Confusion matrices of the CIFAR-10 classification task for the optical neural networks based on NARCA and without RC. **d** Image-denoising performance of the two optical neural networks (with NARCA and without RC) on the BSD68 dataset. The dashed lines with different colors indicate the average denoising performance. **e** Representative denoising results of three sample images obtained using the two optical neural networks

To quantitatively evaluate the effectiveness of the proposed NARCA compare with the traditonal optical convolution operation (without residual connection), the experiments evaluate the performance of two architectures. The architecture schmetics are shown in Fig. 4a. During the transmission of the optical signal, input image information is loaded onto the optical signal via the EOMs. The specific process involves flattening the input image into a one-dimensional vector, which is then encoded in a field-programmable gate array (FPGA). The encoded analog signal is sent to the radio-frequency interface of the EOMs via a 10 GSPS sampling rate digital-to-analog converter (DAC), enabling the time-sequence encoding of specific wavelengths. Since the information transmitted through each channel in NARCA is different (as detailed in Section 3 of the Supplementary Materials), five DAC channels are required to send data streams in parallel, thus modulating five wavelengths simultaneously. The computation results after the image passes through NARCA are detected by the PD, which converts the output into an electrical signal. This signal is then sampled by an analog-to-digital converter (ADC) with a 5 GSPS sampling rate and subsequently read in the FPGA. By re-encoding the residual convolution data and feeding it back into NARCA for further processing, deep optical residual neural networks can be implemented. During the experiments, both neural-network architectures used the same optical chip to perform the convolution operations. All other neural-network parameters were kept identical, including the weight-configuration precision and the input-image precision during computation. In this configuration, the optical hardware is responsible for performing the core convolution operations, while the remaining non-convolutional layers (such as pooling and fully connected layers) are implemented electronically. This hybrid scheme enables a direct, architecture-level comparison between residual and non-residual optical convolution under comparable network depth and model capacity.

**Fig. 5:**
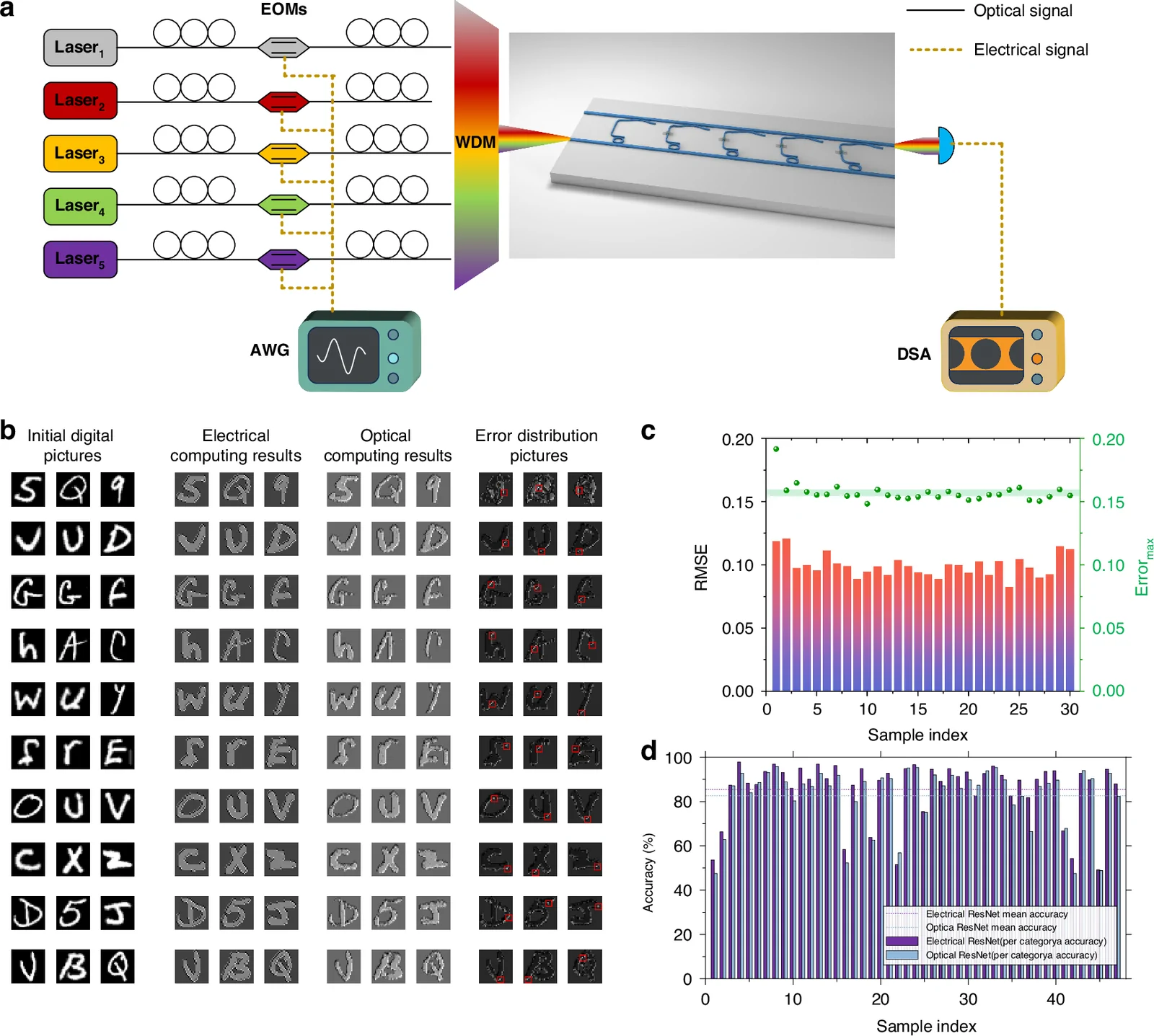
Schematic diagram of the ultrafast optical residual convolution verification system and experimental results. **a** The optical signal and electrical signal transmission process. The result of the input image after AWG encoding is added on the continues optical signal. After the optical residual convolution in NARCA, the result is converted into an electrical signal using a photodetector (PD) and detected by the DSA. The processed feature image can be reconstructed on a host computer, and re-encoded in the AWG to continue residual convolution operations. **b** Comparison of the results of optical residual convolution based on NARCA and traditional electrical residual convolution for the EMNIST dataset, along with the error between the two calculation methods. In the error graph, the red rectangular box highlights the pixel location with the largest error between the two methods. **c** Error statistics between optical residual convolution based on NARCA and traditional electrical residual convolution for 30 sample images. The bar chart shows the RMSE between the two methods for each sample, while the green scatter plot shows the maximum error for each sample. The shaded green region represents the 95% confidence interval of the mean of the maximum errors across all samples. **d** Recognition accuracy of ResNet based on NARCA and a traditional electrical ResNet with the same convolution depth on the EMNIST dataset

Figure 4b, e shows the performance comparison between the NARCA-based residual convolution architecture and the traditional optical convolution architecture across different neural-network tasks. The image classification results on the Cifar-10 dataset are shown in Fig. 4b. The NARCA-based optical residual network reaches an average clasification accuracy of 82%, whereas the optical network without residual connections achieves 65% under the same experimental conditions. This corresponds to a 17% improvement. Figure 5c shows the confusion matrices of the CIFAR-10 classification task for the NARCA-based residual network and the conventional optical convolution network without residual connections. Ten samples per class were evaluated. The results show a higher proportion of correct classifications for the NARCA-based architecture compared with the non-residual architecture. These observations confirm that adding the residual branch leads to a measurable reduction in classification error in this optical computing setting.

We further evaluated the image-denoising performance using the BSD400 dataset, comparing the NARCA-based ResNet-18 with the traditional optical-convolution VGG-18 architecture. The results are shown in Fig. 4d. We selected 20 noisy images for the denoising evaluation using both the NARCA-based architecture and the conventional optical convolution architecture. The structural similarity index (SSIM) values between the denoised images and the corresponding reference images are shown in Fig. 4d. The dashed lines indicate the average SSIM obtained for the two architectures.The distributions of SSIM indicate that, under the same noise conditions, the NARCA-based architecture consistently produces higher SSIM values than the traditional optical-convolution architecture. The average SSIM extracted from these distributions shows an improvement of approximately 0.18 for the NARCA-based model (0.74 of the NARCA-based model and 0.56 of the traditional optical-convolution architecture). This quantitative increase indicates improved reconstruction fidelity and enhanced robustness to input noise. The image-denoising results shown in Fig. 4e further demonstrate the performance advantage of the optical residual convolution mechanism over conventional optical convolution.

### 128GHz Optical residual convolution

To further demonstrate the advantages of NARCA over electronic convolution computing in terms of processing speed and energy efficiency, a 128-GHz high-speed optical residual convolution computing system was experimentally implemented, as shown in Fig. 5a. It should be noted that the 128 GHz experiment demonstrates the achievable operation speed of the optical convolution unit, rather than the end-to-end execution time of a complete neural-network task. The output wavelengths of the five lasers are same with Fig. 3b. The output signals from the five lasers pass through a polarization controller (PC), then enter five EOMs. The output signals from the five EOMs then pass through another PC and are combined into a optical signal via a wavelength-division multiplexer. This optical signal couples into the NARCA chip via an on-chip vertical grating coupler, where it undergoes demultiplexing. On the NARCA chip, convolution results and shortcut connections results are combined, and detected by a photodetector (PD) through another on-chip vertical grating coupler. During the transmission of the optical signal, input image information is loaded onto the optical signal via the EOMs. The specific process involves flattening the input image into a one-dimensional vector, which is then encoded in an arbitrary waveshape generator (AWG). The encoded analog signal is sent to the radio-frequency interface of the EOMs via a DAC with 128 GSPS sampling rate, enabling the time-sequence encoding of specific wavelengths. The computation results after the image passes through NARCA are detected by the PD, which converts the output into an electrical signal. This signal is then sampled by an ADC with a 256 GSPS sampling rate and subsequently read in the digital signal analyzer (DSA). By re-encoding the residual convolution data and feeding it back into NARCA for further processing, deep optical residual neural networks can be implemented.

Figure 5b shows the results of the residual convolution operation using the electrical computation method and our NARCA based on the EMINIST dataset. The first three columns represent the original input digital images, while columns 4 to 6 show the electrical computation results of a single residual convolution. Columns 7 to 9 display the optical computation results of the same residual convolution using NARCA. Columns 10 to 12 show the error distribution between the two computation methods, with the maximum error highlighted in red boxes. All images are displayed with normalization in the range of 0 to 1. From Fig. 5b, it can be qualitatively observed that the error between optical residual convolution using NARCA and traditional electrical residual convolution is minimal, allowing for a good recovery of the electrical computation results and accurate extraction of image features. In Fig. 5b, we quantitatively analyze the computational errors between the two methods. The bar chart in Fig. 5b shows the RMSE between the two computation methods for 30 images, effectively reflecting the statistical error between the two approaches. It can be seen that the RMSE between the two methods is less than 0.125, indicating that the optical residual convolution operation based on NARCA can effectively recover the overall information of the image as computed in the electrical domain.

Additionally, in Fig. 5c, we present the maximum error between the two computation methods for 30 images, as shown by the scatter plot in the figure. It can be observed that the maximum error varies across different images, with the minimum value around 0.15 and the maximum value around 0.19. The larger fluctuations in error may be attributed to system noise caused by jitter in the data sent by the DAC during the experiment. To provide a more reasonable evaluation of the maximum error between the two methods, we plot the 95% confidence interval for the average of the maximum errors, represented by the light green region in Fig. 5b. The upper and lower bounds of the confidence interval are 0.1598 and 0.1542, respectively. The maximum error in the image primarily arises from two factors: first, the jitter in the DAC output signal caused by the DAC encoding precision (which is 3 bits in this work), and second, the imperfect compensation for nonlinear mapping during the photoelectric and electro-optic conversion processes, which leads to some digital signals not being accurately recovered. We will discuss this in more detail in the next section.

However, this maximum error does not significantly impact the image information recovery, as shown in the error map of Fig. 5c. We replace the residual convolution operation in the electrical ResNet-18 neural network with NARCA and calculate the recognition accuracy on the EMNIST dataset. Simultaneously, we perform recognition accuracy calculations for the traditional ResNet-18 neural network under the same network parameters (convolutional layer depth, weight constraints, and number of channels), without using NARCA for the convolution operation. The recognition accuracy results for both neural networks are shown in Fig. 5d. The ResNet-18 based on NARCA has a slight recognition accuracy decreasing (about 4%) on the EMNIST dataset compared to the electrical ResNet-18 neural network. Therefore, NARCA can effectively replace the residual convolution module in electronic ResNet-18 neural networks, thereby promoting the application of optical computing in more complex and generalized neural network models.

In order to quantify the low-power advantage of NARCA in neural network computation, we performed an evaluation of energy efficiency, as shown in section 5 of the Supplementary Materials. NARCA greatly reduces the weight update energy consumption of the chip by utilizing the static zero-power consumption of phase-change material, no external energy is required to maintain the weight state after the weight configuration is finished, and the energy consumption of “0/1” modulation is only on the order of microwatts, which greatly improves the computing efficiency of the optical neural network integrated large-scale synapses. Meanwhile, Table 1 lists a comparison of the relevant parameters, including computational speed, computing efficiency, accuracy, and scalability. The NARCA-based optical ResNet architecture can significantly improve the convolution operation speed and has richer scalability by exploiting the wavelength, polarization, and mode dimensions of light to realize higher computing efficiency.

**Table 1:** Performance comparison of Optical ResNet and electric ResNet

Compute architecture	Computing Speed	Computing Efficiency	Computing accuracy^a^	Scalability
Electric ResNet-18^b^	2.52 GHz	~0.73 TOPS/W	85.73%	Channel
Optical ResNet-18^c^	128 GHz	~0.76 TOPS/W	81.91%	Wavelength, Polarization, Mode, Channel

^a^Range of weights during the training are strictly defined by the unitary performance of NARCA

^b^based on Nvidia 4090

^c^based on NARCA

## Discussion

In summary, we have proposed a method for optical residual convolution acceleration through the NARCA. The obtained results demonstrate that NARCA can replace traditional electronic residual convolution modules, enabling faster and more energy-efficient neural network computations. This marks a significant step toward realizing practical high-performance optical computing systems for AI applications. The experimental results confirm that NARCA-based optical neural network has greater performance compared with the traditional optical neural network. Meanwhile, the deviation (root mean square error, RMSE) between optical convolution based on NARCA and traditional electronic residual convolution is less than 0.125, indicating that optical convolution can almost exactly replicate the results of traditional electronic methods. Furthermore, when compared to the recognition accuracy of the traditional electrical ResNet model on the EMNIST dataset, the ResNet-18 based on NARCA suffers only about 4% accuracy loss. This minimal performance degradation suggests that NARCA can serve as a viable alternative to the electronic residual convolution module without significantly affecting the overall network performance.

There are two main factors that might explain the slight decrease in accuracy. Firstly, the weight levels of the optical synapse on NARCA do not exhibit the ideal linear variation. As PCMs approach their crystalline state, they show a stronger absorption effect on optical pulses, requiring less energy for optical pulses. However, in practical applications, the weight changes in the neural networks are often nonlinear, which contributes to some computational error. This nonlinearity could potentially be mitigated by more refined pulse encoding to compensate for the absorption behavior of all-optical synapses. Secondly, errors might arise from the jitter of the DAC during data transmission and the nonlinear transformations during system calibration. These issues could be resolved in future work through optimized calibration techniques.

Meanwhile, due to the inherent wavelength sensitivity and limited fabrication tolerance of MRRs in NARCA, achieving large-scale integration using MRRs requires careful design of their quality factor (Q-factor) to balance spectral selectivity and fabrication robustness. MRRs with lower Q-factors exhibit broader resonance linewidths, which improves tolerance to small wavelength shifts while maintaining sufficient modulation contrast. However, this also limits the capacity for dense WDM. In practical large-scale integration, it is nearly impossible for the resonance wavelengths of an MRR array to perfectly align with the original design due to fabrication-induced variations. Therefore, post-fabrication tuning is essential to align the resonances precisely. One promising approach is to integrate PCMs onto the MRRs, enabling non-volatile tuning of the resonance spectra. This method allows for permanent correction of resonance mismatch without the need for continuous electrical or thermal power, making it highly suitable for large-scale photonic neural network implementations.

Moreover, we believe that frequent opto-electronic and electro-optic conversions severely limit the computational speed and energy efficiency of residual neural networks based on NARCA. A more effective solution would involve integrating optical and microelectronic components. By integrating high-sensitivity PD at the output port of NARCA and designing nonlinear electronic response devices that meet the required specifications, a complete residual computation could be achieved. This would significantly reduce the time and energy required for signal processing and encoding in the subsequent electronic domain. Such developments could unlock a new era of high-performance, energy-efficient AI computations, particularly in large-scale, data-intensive tasks. The integration of NARCA with existing microelectronics could serve as a bridge for transitioning optical computation into more general-purpose, flexible, and scalable AI applications.

## Materials and methods

### NARCA fabrication

The NARCA is fabricated on the SOI platform with a 220-nm-thick silicon top layer and a 2-μm-thick buried oxide. The fabrication process begins with the silicon waveguide structure for NARCA using electron beam lithography (EBL) exposure followed by inductively coupled plasma (ICP) etching. This step defines the waveguide pattern on the SOI substrate, which serves as the foundation for the optical components of the accelerator. Subsequently, EBL is employed to pattern the areas on the NARCA where PCMs need to be deposited. Once the desired pattern is defined, direct current (DC) magnetron sputtering is used to deposit 25 nm and 20 nm thick layers of GST (Ge_2_Sb_2_Te_5_) and indium tin oxide (ITO) thin films, respectively. After deposition, the lift-off process is carried out to remove unwanted material, leaving behind the patterned NARCA structure.

### Experimental setup

Weight Configuration: The weight configuration of NARCA is controlled by a pulsed laser (FL-1550-AO-20uJ-FG70888) combined with a tunable laser (Santec TSL-710) through a 9:1 beam splitter. The laser pulses, with specific energy and pulse width, adjust the weights on NARCA.

## Supplementary information

Supplementary Information for On-chip non-volatile all-optical residual neural network accelerator

## Data Availability

The data that support the findings of this study are available from the corresponding author upon reasonable request.
