# Supplementary material for: On-chip non-volatile all-optical residual neural network accelerator
Source: Light Sci Appl. 2026 Aug 19;15:348. doi: 10.1038/s41377-026-02325-2 (PMC13490518; doi:10.1038/s41377-026-02325-2)
Supplement: Supplementary file 1 [file 41377_2026_2325_MOESM1_ESM.docx]

Supplementary Information for

**On-chip non-volatile** **all-optical residual neural network accelerator**

Zhiqiang Quan^1,2,3,#^, Bing Han^1,2,3,#^, Xiaoxiao Ma^1,2,3^, Jingyuan Qu^4,5^, Xiangshui Miao^4,5^, Qiang He^4,5*^, and Jian Wang^1,2,3,^^*^

*^1^Wuhan National Laboratory for Optoelectronics and School of Optical and Electronic Information,* *Huazhong University of Science and Technology, Wuhan 430074, Hubei, China*

*^2^Optics Valley Laboratory, Wuhan 430074, Hubei, China*

*^3^Optics Valley Laboratory, Wuhan 430074, Hubei, China*

*^4^School of Integrated Circuits, Huazhong University of Science and Technology, Wuhan 430074, Hubei, China*

*^5^Hubei Yangtze Memory Laboratories, Wuhan 430205, Hubei, China*

\# These authors contribute equally to this work.

*** *Corresponding author:* [*qianghe@hust.edu.cn*](mailto:qianghe@hust.edu.cn)*,* [*jwang@hust.edu.cn*](mailto:jwang@hust.edu.cn)

The Supplementary Material includes:

[Section 1: Design of Waveguide Structures	3](#_Toc200121190)

[Section 2: Design of Non-Volatile Optical Synapses	5](#_Toc200121191)

[Section 3: Multi-Channel Data Encoding for Residual Convolution	8](#_Toc200121192)

[Section 4: Dataset and Neural Network Training	11](#_Toc200121193)

[Section 5 Compared with other technology	11](#_Toc200121194)

[Section 6: Fabrication flow schematic of NARCA	13](#_Toc200121195)

[Reference	14](#_Toc200121196)

# Section 1: Design of Waveguide Structures

**Design of Micro-Ring Resonators**

In the optical residual neural network, five distinct wavelengths are required to encode the shortcut connection data stream and the convolution data stream. Therefore, we designed five micro-ring resonators with different resonant wavelengths to achieve wavelength division multiplexing (WDM). Considering the inevitable fabrication errors, the resonant wavelengths of the micro-ring resonators may deviate from their designed values, and the all-optical modulation process does not allow for post-fabrication calibration of these wavelengths. To ensure sufficient channel isolation between the different wavelength signals, we selected five working wavelengths within a sufficiently large free spectral range (FSR). A larger FSR typically requires a smaller micro-ring radius. However, this comes at the cost of increasing the loss coefficient of the micro-ring resonators. Balancing the trade-offs between FSR, fabrication tolerances, and resonator losses is critical for achieving optimal performance in the optical residual convolution operation. In this work, we utilized bent couplers in the coupling region to balance the losses of the micro-ring resonators and achieve a larger FSR. Additionally, the width of the micro-ring was reduced in the bent coupling region to increase the coupling strength between the bent waveguide and the micro-ring. The advantage of this approach is that it allows for a higher coupling strength even when the gap between the micro-ring and the bent waveguide is relatively large, thereby reducing the stringent requirements on fabrication processes [1]. To minimize bending losses of light in the micro-ring, we selected a micro-ring width of 650 nm to reduce the bending loss of the quasi-TE mode during propagation. At the midpoint of the bent coupling region, the micro-ring width was reduced to 500 nm to enhance coupling strength with the bent waveguide, which maintained a constant width of 380 nm. As a result, the designed micro-ring resonator is an elliptical micro-ring resonator with an initial semi-major axis radius of 3.009 µm and a semi-minor axis radius of 2.009 µm. The radius of the other four micro-ring resonators, which support different resonant wavelengths, were incrementally increased by 17 nm. The gap between the bent waveguide and the micro-ring remained constant at 180 nm. The SEM image of the designed micro-ring resonator is shown in Fig. S1a. The experimentally measured transmission spectra of the five micro-ring resonators with different radii are presented in Fig. S1b.

Figure S1. (a) Elliptical micro-ring resonator with a large FSR. (b) Transmission spectra of five elliptical micro-ring resonators with different radii, showing an FSR of approximately 34 nm.

**Design of Broadband Directional Coupler (BDC)**

In the optical residual neural network schematic shown in Fig. 1, the optical signals transmitted in five different channels need to be coupled into a single output waveguide to achieve addition operations. Since each channel transmits a distinct wavelength, the directional coupler must possess broadband characteristics. Additionally, the number of BDCs traversed by each channel varies, and due to the reciprocal nature of coupling, the transmission loss is highest for the wavelength *𝜆*_0_ transmitted through the first channel. To ensure that each wavelength can be detected by the downstream photodetector, the on-chip loss needs to be carefully optimized, particularly controlling the transmission loss for the wavelength in the first channel. Assuming that the insertion loss of the directional couplers is negligible, the total loss for the wavelength *𝜆*_0_ in the first channel after passing through five directional couplers can be expressed as:

$$LOSS\text{=-10lg}\left[\kappa(1-\kappa)^{4}\right]$$

Here, 𝜅 represents the intensity attenuation coefficient of the broadband coupler. When 𝜅 is restricted to a range of 0.1–0.9, calculations indicate that the transmission loss of the first channel is minimized when *𝜅*=0.2. Therefore, a broadband directional coupler with an intensity attenuation coefficient of 0.2 (coupling coefficient of 0.8) is required, designed to operate over the C-band. Typically, directional couplers face challenges in achieving a wide operating bandwidth due to wavelength dispersion. However, by introducing curvature-induced dispersion in the device, the wavelength-induced dispersion can be balanced, thereby achieving a broader bandwidth [2]. We designed a broadband directional coupler as shown in Fig. S2a, with a waveguide width of approximately 400 nm and a waveguide gap of about 175 nm. The splitting performance is illustrated in Fig. S2b. In Fig. S2b, it can be observed that the splitting performance of the bent directional coupler in the C-band deviates by a maximum of approximately 10% from the design target. This deviation arises because the fabricated waveguide gap is slightly larger than the designed 150 nm. Nevertheless, the coupler can still effectively combine five different wavelengths into a single output channel.

Figure S2. (a) SEM image of the bent directional coupler, with a waveguide width of approximately 400 nm, a waveguide gap of around 175 nm, a bending radius of approximately 28 µm, and a sector angle of about 33 degrees. (b) Broadband splitting performance of the bent directional coupler.

# Section 2: Design of Non-Volatile Optical Synapses

**Optical Simulation of Non-Volatile Synapses**

The non-volatile optical synapse designed in this study is a loss-modulation optical synapse based on the refractive index modulation of GST. The length of the GST has a significant effect on the switching ratio of the optical synapse, as theoretically a longer length of phase-change material leads to a higher switching ratio. However, due to the high loss coefficient of GST in the amorphous state, the length of the GST also contributes to the insertion loss of the optical synapse [3]. To balance the switching ratio and insertion loss of the optical synapse, an appropriate GST modulation length is selected based on optical simulations using the finite-difference time-domain (FDTD) method. In the simulations, the refractive indices of the GST thin film at 1550 nm wavelength are set to 4.28+0.11i in the amorphous state and 6.68+0.97i in the crystalline state, and the refractive indices of the ITO thin film at 1550 nm is 1.93. A 25 nm thick GST film was deposited on a silicon waveguide to form the non-volatile optical synapse, and a 20 nm thick ITO film used to protect the GST from oxidation. The silicon waveguide is a single-mode waveguide with a width of 500 nm and a height of 220 nm, supporting quasi-TE mode transmission. By varying the GST length along the light propagation direction, the trends of insertion loss and extinction ratio for the non-volatile optical synapse were obtained, as shown in Fig. S3. To balance on-chip losses and the device's switching ratio, a GST deposition length of 5 µm was selected. This length ensures sufficient switching performance while minimizing excess loss introduced by the phase-change material.

Figure S3 Relationship between the insertion loss and extinction ratio of the non-volatile optical synapse and the deposition length of the phase-change material.

**Thermal Simulation of Non-Volatile Optical Synapse**

To investigate the feasibility of modulating non-volatile optical synapses using optical pulses and to determine the specific parameters of the optical pulses, we perform optical-thermal coupled simulations based on the finite element method (FEM). The geometric and optical parameters of the waveguide, phase-change material, and ITO thin film are consistent with those used in the insertion loss and extinction ratio simulations of the non-volatile optical synapses. The thermodynamic parameters are summarized in the Table S1[4,5]. By transmitting optical pulses of specific pulse widths in the waveguide, the temperature of the phase-change material is increased via the material’s optical absorption effect, inducing a change in its crystalline state. The pulse widths are consistent with those used in experiments: 150 ns for crystallization and 10 ns for amorphization. Fig. S4a and Fig. S4b shows the mode distribution on the cross-section of the non-volatile optical synapse perpendicular to the light propagation direction when the GST is in amorphous state and crystalline state, respectively. It can be observed that the optical field energy is concentrated in the region of the phase-change material, which has a higher refractive index. Using the thermodynamic parameters of the material, the temperature distribution of the non-volatile optical synapse can be calculated. Fig. S4c and d illustrates the stable temperature distribution of the non-volatile optical synapse at a crystallization pulse energy of 3 dBm, and an amorphization pulse energy of 10 dBm. The temperature distribution indicates that the maximum temperature is located in the phase-change material region. This is due to the phase-change material having higher optical absorption coefficient and lower thermal conductivity compared to silicon and ITO materials. The average temperature of the GST thin film reaches 360.12 K and 603.57 K, respectively.

Table S1 Thermal properties of the materials used in the FEM simulation

| Material | Thermal conductivity (W m^-1^ K^-1^) | Heat Capacity  (J kg^-1^ K^-1^) | Density  (kg m^-3^) |
| --- | --- | --- | --- |
| a_GST | 0.19 | 213 | 5870 |
| c_GST | 0.57 | 199 | 6270 |
| ITO | 11 | 1290 | 7100 |
| Si | 149 | 720 | 2329 |
| SiO_2_ | 1.38 | 740 | 2203 |

Figure S4. Optical and thermal simulation of the non-volatile optical synapse. Normalized electric field distributions of the non-volatile optical synapse when GST is in amorphous state (a) and crystalline state (b). Temperature field distributions in the central cross section of the non-volatile optical synapse under external optical pulse excitation when the GST is in amorphous state (c) and crystalline state (d).

# Section 3: Multi-Channel Data Encoding for Residual Convolution

In optical residual convolution, input image data must undergo rigorous encoding to enable convolution and residual operations in the optical domain. Taking handwritten digit images as an example (28×28 pixel images from the EMNIST dataset), the encoding process begins with normalizing the image data to adjust pixel values to a range of 0 to 1, ensuring compatibility with the dynamic range of the optical system. To maintain consistent dimensions between the convolution signals and the shortcut connection signals, zero-padding is applied to the right and bottom edges of the convolution signal matrix, maintaining a 28×28 matrix size. This ensures that with a convolution stride of 1 and a kernel size of 2×2, the output dimensions of the residual convolution match the original 28×28 matrix, consistent with the dimensionality in electronic convolution processes. During optical encoding, the image data is distributed across multiple optical channels, each responsible for specific data tasks. The shortcut connection channel encodes the entire 28×28 original image, mapping each pixel's normalized grayscale value directly to optical intensity. The four convolution-related channels are assigned to each pixel in the convolution kernel region. For example, the top-left, top-right, bottom-left, and bottom-right pixels of the kernel are encoded into Channels 2, 3, 4, and 5, respectively. As the convolution kernel slides across the image, these channels carry a series of time-sequenced optical intensity signals that are combined with convolution kernel weights to complete the convolution operation, as illustrated in Fig. S5. The core of optical convolution lies in multiplying input signals with preset convolution kernel weights and summing the results. For each convolution region, the optical intensity signals carried by Channels 2 to 5 are multiplied by their corresponding weights in the weight matrix, with the results accumulated in the optical domain to form the convolution output signal. Subsequently, the convolution result is added to the shortcut connection signal in the same optical path to generate the final residual convolution output signal. During encoding, synchronization across all channels is crucial to ensure that the convolution signals and shortcut connection signals are strictly aligned in time, thereby maintaining the correctness of the residual operations. The encoded data is then converted into analog voltage signals via the DAC. The initial digital signals for Channels 1–5 and their corresponding time-sequenced voltage signals are shown in Fig. S6. From the figure, it is evident that the time-sequenced voltage signals output by the DAC for each channel closely match the trends of the original digital signals, demonstrating effective restoration of the encoded image data.

Figure S5. Optical residual coding with a 6×6 input image as an example.

Figure S6. Comparison between DAC timing voltage information and initial digital information of different signal channels during residual convolution process.

# Section 4: Dataset and Neural Network Training

The EMNIST (Extended MNIST) dataset is an extended version of the MNIST dataset, specifically designed for handwritten character recognition tasks. For this study, we selected 47 classes of characters from EMNIST, including digits (0–9) and a subset of uppercase (A-Z) and lowercase letters (a, b, d, e, f, g, h, n, q, r, t). Each character is represented as a 28×28 grayscale image. During data preprocessing and training, the input images were encoded using 3-bit grayscale encoding to fit the dynamic range limitations of optical computations. The weights were encoded with 5-bit precision, matching the weight levels of the all-optical synapses. This encoding ensures the feasibility and precision of optical convolution operations, where each weight level corresponds to a finely tunable change in optical intensity within the synapse, enabling efficient mapping of the input signals. For training, we employed stochastic gradient descent (SGD) with a learning rate of 0.005 as the optimizer. SGD updates model parameters iteratively to minimize the loss function, and a carefully chosen learning rate accelerates model convergence while preventing instability caused by excessively large updates. ReLU (Rectified Linear Unit) was used as the nonlinear activation function, effectively introducing nonlinearity to the model by truncating negative values. This choice also mitigates gradient vanishing issues, enhancing both training efficiency and the model’s expressive capacity. Under this configuration, the combination of carefully designed weight and input encoding allowed the model to optimize performance on the EMNIST dataset while adapting to the characteristics of optical computing. This setup lays a solid foundation for subsequent residual neural network training and real-world applications. In the actual training process, the time required for 100 iterations of the neural network is about 30 min.

# Section 5: Compared with other technology

To further evaluate the advantages of NARCA in neural network computing, this section presents the key performance metrics of NARCA in detail. In the system-level experiment, we performed optical residual convolution computations at an equivalent signal frequency of 128 GHz. The relationship between the computation results and the input signals is given in Equation (1) of the main manuscript. Since one optical residual computation involves four real multiplications and four real additions, the total number of operations performed by NARCA at a 128 GHz equivalent signal frequency is:

$$128 \mathrm{GHz}\times8=1 \mathrm{TOPS}$$

The device footprint of NARCA is 3 × 0.5 mm², from which the computing density can be calculated as:

$$\frac{128 \mathrm{GHz}\times8}{{3\times0.5 \mathrm{mm}}^{2}}=0.67 TOPS/\mathrm{mm}^{2}$$

Through layout optimization, the computational density can be enhanced by more than an order of magnitude (i.e., over 10× improvement). Meanwhile, considering the energy efficiency advantages of non-volatile materials—namely, the elimination of the need for continuous power to maintain the weight states—the computing efficiency of NARCA is expected to significantly outperform other neural network architectures based on thermo-optic phase shifters or MRRs. The computing efficiency is defined as:

$$Computing efficiency=\frac{Computing speed (\mathrm{TOPS})}{Power consumption (W)}$$

In NARCA, on-chip weight updates are achieved through four non-volatile optical synapses. The maximum peak pulse energy for the amorphous-to-crystalline transition is 17.1 dBm with a pulse width of 150 ns, while the crystalline-to-amorphous transition requires a maximum peak pulse energy of 29.9 dBm with a pulse width of 10 ns. Accordingly, the average energy required per unit time for the amorphization and crystallization processes can be calculated as follows:

$$\begin{aligned} P_{cry}={10}^{\frac{17.1}{10}}\times\frac{150 \mathrm{ns}}{1 s}\approx7.69\times{10}^{-6}W \\ P_{amo}={10}^{\frac{29.9}{10}}\times\frac{10 \mathrm{ns}}{1 s}\approx9.77\times{10}^{-6} W \end{aligned}$$

The above two energy consumptions correspond to the "1-to-0" and "0-to-1" modulation processes, respectively. By selecting the higher energy consumption associated with the "0/1" modulation as a reference, the weight update energy consumption of NARCA is estimated to be approximately 9.77×10^-6^ W. Considering during the computing process, NARCA don’t need external energy to remain the weight strength, the computing energy consumption only include signal source (laser), signal encoding (DAC) and signal detection (ADC).

(1) The laser used in our experiment is a TTX1995 with a power consumption of approximately 2.5 W.
(2) For optical signal generation, the DAC-based transmitter in [6] achieves an energy efﬁciency of 2.8 pJ per Baud, while the modulator achieves an energy efﬁciency of 42 fJ per Baud [7]. Additionally, the modulator bias consumes approximately 16 μW for π shift. Therefore, the combined energy consumption of the DAC (operating at a sampling rate of 128 GSamples/s) and the modulator is approximately 0.364 W.
(3) The energy consumption of EDFA is estimated using the formula$\frac{\lambda_{S}}{\lambda_{P}}(P_{out}^{S} - P_{in}^{S})/\eta$ [8], where the signal central wavelength is about λs = 1543 nm, the pump wavelength is $\lambda_{P}$ = 1480 nm, the output power is $P_{out}^{S}$ ≈ 20 dBm, the input power is $P_{in}^{S}$ ≈ 0 dBm, and the wall- plug efficiency is η ≈ 0.3. Based on these parameters, the energy consumption of EDFA is approximately 0.34 W.
(4) For optical signal detection, the ADC-based receiver achieves an energy efficiency of 2.82 pJ
    per Baud [4], while the photodetector achieves an energy efficiency in the range of a few fJ per bit [5]. Therefore, the total energy consumption of the ADC (operating at a sampling rateof 128 GSamples s^-1^) and the photodetector is approximately 0.361 W.

Moreover, since the non-volatile optical synapses in NARCA do not require energy to maintain their weight states during computation, NARCA offers greater advantages for neural network tasks involving long computation times and large-scale optical neural network which need great amount of optical tunable synapse. For example, assume a 10×10 cross-bar architecture, it can perform 90 multiplication operations and 100 addition operations simultaneously at each computing cycle. Combining the energy consumption of all components—laser, DAC, modulator, ADC, and photodetector—the total energy consumption of the all-optical engine is approximately 32.59 W. Therefore, the computing efficiency can be expressed as:

$$Compute efficiency=\frac{128Hz \times190}{32.59 W}\approx0.75 TOPS/W$$

The table below compares the key parameters between NARCA and conventional volatile optical convolution architectures:

Table S2: Comparison of photonic computing hardware

| Compute architecture | Clock speed (GHz) | Weight update energy consumption  (W) | Compute efficiency ^a^  (TOPS/W) | Ref. |
| --- | --- | --- | --- | --- |
| NARCA ^a^ | 128 | 9.77×10^-4^ | 0.75 |  |
| MZI convolution | 10 | 2.7 | 0.44 | [9] |
| MRR convolution | 17 | 0.24 | 0.2 | [10] |
| MZI convolution | 60 | 0.011 | 2.39 | [11] |
| MZI convolution | 43.8 | NA | 17.4 | [12] |
| MRR convolution | 30.67 | 0.61 | 0.27 | [13] |

a: Based on our proposed NARCA which includes 10 channel parallel residual convolution.

# Section 6: Fabrication flow schematic of NARCA

Figure S7. The Fabrication flow schematic of NARCA.

# References

1. Tan, Y. et al. Polarization-selective microring resonators, *Optics Express*, **25**, 4106-4119 (2017).
2. El-Saeed, A. H. et al. Low-Loss Silicon Directional Coupler With Arbitrary Coupling Ratios for Broadband Wavelength Operation Based on Bent Waveguides, *Journal of Lightwave Technology* **42**, 6011-6018 (2024).
3. Zhang, H. et al. On-Chip Photonic Synapses Based on Slot-Ridge Waveguides With PCMs For In-Memory Computing, *IEEE Photonics Journal* **13**, 1-13 (2021).
4. Zhang, M. et al. Nonvolatile Multilevel Switching of Silicon Photonic Devices with In2O3/GST Segmented Structures. *Advanced Optical Material* **11**, 2202748 (2023).
5. Xia J. et al. Seven Bit Nonvolatile Electrically Programmable Photonics Based on Phase-Change Materials for Image Recognition. *ACS Photonics* **11**, 723-730 (2024).
6. Kossel, M. A. et al. 8.3 an 8b DAC-based SST TX using metal gate resistors with 1.4 pj/b efficiency at 112Gb/s PAM-4 and 8-Tap FFE in 7nm CMOS. 2021 *IEEE International Solid-State Circuits Conference (ISSCC)* **64**, 130–132 (2021).
7. Wang, C. et al. Integrated lithium niobate electro-optic modulators operating at CMOS-compatible voltages. *Nature* **562**, 101–104 (2018).
8. Feng, H. et al. Integrated lithium niobate microwave photonic processing engine. *Nature* **627**, 80–87 (2024).
9. Bandyopadhyay, S. et al. Single-chip photonic deep neural network with forward-only training. *Nature Photonics* **18**, 1335-1343 (2024).
10. Bai, B. et al. Microcomb-based integrated photonic processing unit. *Nature Communications* **14,** 66 (2023).
11. Lin. Z. et al. 120 GOPS Photonic tensor core in thin-film lithium niobate for inference and in situ training. *Nature Communications* **15**, 9081 (2024).
12. Hu, Y. et al. Integrated lithium niobate photonic computing circuit based on efficient and high-speed electro-optic conversion. *Nature Communications* **16**, 8178 (2025).
13. Meng, X. et al. High-integrated photonic tensor core utilizing high-dimensional lightwave and microwave multidomain multiplexing. *Light: Science & Applications* **14**, 27 (2025).
